# Does negative marriage discourse on social media deter marriage? Evidence from Weibo users in urban China

**DOI:** 10.3389/fpsyg.2026.1902706

**Published:** 2026-09-04

**Authors:** Ye Chen, Lyuci Zhang, Yuqin Jiang, Zeinab Zaremohzzabieh, Aini Azeqa Ma’rof

**Affiliations:** 1 College of Education, Beibu Gulf University, Qinzhou, Guangxi, China; 2 Faculty of Teacher Education, Hezhou University, Hezhou, China; 3 Women and Family Studies Research Center, University of Religions and Denominations, Qom, Iran; 4 Institute for Social Science Studies, Universiti Putra Malaysia, Serdang, Selangor, Malaysia

**Keywords:** China, elaboration likelihood model, marriage attitude, marriage intention, social media, theory of planned behaviour, unmarried women, Weibo

## Abstract

**Introduction:**

Marriage-related discourse on social media has become a prominent source of social influence, yet its impact on women’s marriage-related cognitions in contemporary China remains underexplored. This study integrates the Elaboration Likelihood Model (ELM) and the Theory of Planned Behaviour (TPB) to examine how characteristics of Weibo marriage comments influence marriage attitudes and intentions among unmarried Chinese women.

**Methods:**

A cross-sectional survey was conducted with 373 unmarried women in Guangzhou, China. The proposed research model incorporated ELM constructs (argument quality, source credibility, and personal relevance) and TPB constructs (marriage attitude, subjective norms, perceived behavioural control, and marriage intention). Data were analysed using partial least squares structural equation modelling (PLS-SEM).

**Results:**

Both argument quality and source credibility were negatively associated with marriage attitude and marriage intention. Marriage attitude served as a complementary mediator in these relationships, indicating that online comment characteristics influence intention both directly and indirectly via attitudinal evaluations. Personal relevance moderated the effects of comment characteristics on marriage attitude, suggesting that women with higher self-relevance were more responsive to online cues. Additionally, subjective norms, marriage attitude, and perceived behavioural control positively predicted marriage intention.

**Discussion:**

These findings extend theoretical understanding of the psychological processes through which social media discourse shapes marriage-related decision-making. They also highlight the role of digital environments in influencing personal attitudes and intentions. The study offers practical implications for clinicians and counselors supporting unmarried women navigating relationship beliefs and marriage-related concerns in digitally mediated contexts.

## 1 Introduction

The modernization of China has also been coupled with massive demographic transformation, with a decline in marriage and fertility rates (Zhang et al., 2025). The gross marriage rate in China declined to 4.8 per thousand in 2022, indicating a substantial change in marriage attitudes and intentions among young adults (Zhong et al., 2024). This trend is not specific to China but represents part of a broader East Asian pattern of declining marriage, driven by rapid economic development, rising educational attainment, and changing gender norms that contribute to delayed or avoided marriage (Raymo et al., 2015). This transformation is particularly evident in China, where unmarried women have gained greater autonomy in decisions regarding marriage and childbearing due to increased education, professional development, and economic independence (Gui, 2020). However, this expansion of individual autonomy has occurred alongside the continued influence of traditional family values, which emphasize early marriage, family obligations, and gendered expectations regarding women’s roles. The coexistence of these competing value systems creates tensions in women’s marriage decision-making and generates social pressures surrounding whether, when, and why women should marry (Zhong and Wilkinson, 2025). Marriage attitudes and intentions are shaped by broader demographic, economic, and sociocultural changes and, in turn, have important implications for population structure and social stability. Accordingly, understanding the factors that influence unmarried women’s marriage attitudes and intentions has become increasingly important for informing evidence-based social and population policies.

The Theory of Planned Behaviour (TPB; Ajzen, 1991) conceptualises intention as an outcome of attitudes, subjective norms, and perceived behavioural control. However, treating intention as an outcome does not reduce its importance in explaining behavioural decision-making. Rather, intention is the most immediate antecedent of behaviour, reflecting individuals’ willingness and commitment to translate their evaluations and perceived constraints into concrete behavioural plans (Fishbein and Ajzen, 2011). Accordingly, intention should not be viewed as an extension of attitudes or norms but as a distinct motivational construct that links psychological evaluations to behavioural readiness. This perspective is further supported by Miller's (1994) Traits–Desires–Intentions–Behaviour (TDIB) model, which identifies intention as a key transitional stage between motivational traits and behavioural outcomes. Within the TDIB framework, intentions are deliberate behavioural commitments formed after individuals evaluate their desires in light of practical constraints, including economic resources, occupational demands, health status, and social expectations (Miller et al., 2004). Therefore, intentions represent more than general preferences; they reflect individuals’ judgments about whether their desired behaviours are realistic and achievable within their personal and social circumstances.

This perspective is consistent with demographic research, which distinguishes general preferences from behavioural intentions by recognising that intentions incorporate feasibility assessments and contextual constraints that are more directly related to subsequent behaviour (Testa, 2014; Philipov et al., 2015). Likewise, research on marriage decision-making identifies intention as a distinct motivational mechanism linking individual evaluations and social influences to behavioural outcomes (Bachrach and Morgan, 2013). Accordingly, marriage intention is conceptualised in the present study as a separate motivational construct rather than as a dimension of attitudes, subjective norms, or perceived behavioural control. Instead, it represents the immediate behavioural commitment through which marriage-related evaluations are translated into behavioural readiness. Because marriage intentions develop through the integration of individual evaluations, social influences, and contextual information, understanding how such information is acquired and psychologically processed is essential for explaining marriage intention formation.

Social media has turned into an effective instrument of attitudinal transformation in modern society (Valkenburg et al., 2016). Weibo, a microblogging site that has more than 580 million monthly active users, is the high profile public forum in China where issues related to marriage are widely discussed. Being a unique Chinese social media platform, Weibo allows posting, reposting, and commenting on marriage-related matters and, as a result, an interactive information space is formed, where a new discourse on marriage is constantly produced and negotiated publicly (Shao and Wang, 2017). For unmarried women, Weibo provides a relatively accessible space to express personal experiences, discuss perceived social pressures, and negotiate the tension between traditional marriage expectations and changing gender identities. Therefore, women’s marriage-related expressions on Weibo are not simply individual opinions but also reflections of broader social conflicts between established gender norms and increasing female autonomy (Guo, 2025).

The following are some of the most common marriage-related remarks on Weibo that relate to gender inequality, financial strains of marriage, domestic violence, perceived incompatibility of marriage with career progression, and the emotional cost involved in marital relationships. Interestingly, studies indicate that the negativity prevails in the discourse related to marriage in Chinese social media, which sets the information field where negative attitudes toward marriage are disproportionately represented and shared (Cao, 2025). These are user-created remarks, which form a major negative persuasion information space that can greatly influence the views of marriage among the unmarried women. Studies of online information processing have repeatedly shown that negative information has disproportionately powerful effects on attitudes and behavioural intentions than positive information- a phenomenon which is due to the negativity bias (Qahri-Saremi and Montazemi, 2023). Thus, there is a necessity to investigate the psychological processes by which marriage attitudes and intentions of unmarried women can be affected by Weibo marriage comments in a systematic manner.

Research into changes in marriage attitudes has been primarily focused mainly on macro-level changes in attitudes or the basic communication characteristics of social media platforms (Cherukut et al., 2025). While the trajectories of marriage attitudes and intentions from both the sociological and demographic perspectives have been studied to some extent, the impact of social media comments on people’s attitudes toward marriage has received little attention, especially regarding the psychological processes involved (Wang et al., 2025). This is especially true in the Chinese context, where relationships between the values of marriage and family in Confucianism, both of which have social and psychological elements of attitudes, and modern individualism creates an environment of sociocultural contrasts on attitudes (Zhong and Wilkinson, 2025). Additionally, although previous studies have examined marriage intention in relation to personal attitudes, social norms, and perceived behavioural control, there remains a lack of an integrated framework explaining how social media information is psychologically processed and translated into marriage intention among unmarried women.

To address this gap, this study combines the Elaboration Likelihood Model (ELM) and the TPB (Petty and Cacioppo, 1986; Ajzen, 1991). The ELM illustrates the impact of argument quality and source credibility in a persuasive message. ELM explains that persuasive information is processed through two routes. The central route involves careful evaluation of message content, whereas the peripheral route relies on external cues such as source credibility. In this study, argument quality represents the central route, while source credibility represents the peripheral route, explaining how Weibo marriage comments influence unmarried women’s marriage attitudes.

The TPB explains how attitudes, subjective norms, and perceived behavioural control jointly shape behavioural intention, which is the immediate antecedent of behaviour (Ajzen, 1991). In contrast, the ELM explains how persuasive information influences attitude formation through central and peripheral processing routes. Marriage attitude provides the conceptual bridge between these theories by linking the psychological processing of social media information to the formation of marriage intention. Integrating the ELM and TPB therefore enables a comprehensive explanation of how persuasive online information shapes marriage attitudes and how these attitudes subsequently contribute to marriage intention. Although this integrated framework has been widely applied in consumer behaviour, technology acceptance, and health communication, its application to marriage decision-making remains limited (Cyr et al., 2018; Pillai and Sivathanu, 2020; Ragab, 2022).

In particular, this study has five research goals: (1) elucidate the relationship between argument quality, source credibility, and marriage attitude; (2) elucidate the relationship between argument quality, source credibility, and marriage intention; (3) elucidate the relationship between marriage attitude, subjective and descriptive norms, perceived behavioral control (PBC), and marriage intention; (4) assess the effect of marriage attitude as a mediator on the relationship between argument quality, source credibility and marriage intention; and (5) assess the effect of personal relevance as a moderator on the relationship between argument quality, source credibility and marriage attitude.

In this study, there are three major contributions. Firstly, it extends the application of the ELM and TPB to marriage decision-making by integrating them into a unified framework that explains how social media information processing shapes marriage attitudes and subsequently influences marriage intention. Secondly, it fills the empirical void on the specific psychological processes that Weibo comments use to shape marriage-related thinking for the Chinese unmarried female. Finally, the findings provide practical implications for the development of evidence-based marriage and population policies and for enhancing government communication and information management on social media.

## 2 Literature review and hypotheses development

### 2.1 Marriage attitude and social media influence

Marriage attitude refers to an individual’s overall evaluative tendency toward the prospect of getting married, encompassing favourable or unfavourable judgments (Ajzen, 1991). As a core construct in the TPB, attitude is conceptualised as a learned predisposition to respond consistently toward a given object, shaped by an individual’s salient beliefs about the consequences of performing a behaviour (Fishbein and Ajzen, 2011). In the context of marriage, these beliefs encompass expected outcomes related to emotional fulfilment, financial security, personal autonomy, and social status, which collectively determine whether marriage is evaluated positively or negatively.

Prior research has identified several antecedents of marriage attitude. At the individual level, educational attainment, career orientation, and economic independence have been associated with more cautious or negative evaluations of marriage, particularly among women (Ji, 2015; Gui, 2020). At the social level, family expectations, peer norms, and cultural values shape evaluative frameworks within which marriage is assessed (Koçak and Mouratidis, 2024). More recently, scholars have begun to examine informational antecedents—specifically, how exposure to marriage-related content in media environments influences attitude formation (Banjo, 2002; Cherukut et al., 2025).

The rise of user-generated content (UGC) platforms has introduced a new category of informational influence on marriage attitudes. Research has demonstrated that UGC can significantly shape attitudes and behavioural intentions by providing socially endorsed, experiential information that audiences perceive as more authentic than institutional messaging (Rim and Song, 2016). Importantly, the persuasive impact of UGC is not uniform across audiences: women have been found to be particularly responsive to opinion-based social media content when forming attitudes toward interpersonal and relational domains (Vijayalakshmi and Bhattacharyya, 2012).

Marriage-related discourse on Weibo is marked by elevated levels of engagement, a strong emotional appeal, and a highly critical tone in particular (Li and Zhou, 2025; Ma, 2025). The literature on marriage discourse on Chinese social media has, however, been doing content-analytic or discourse-analytic studies, which report on what is said about marriage without analyzing how this content has a psychological impact on its audience (Yuan, 2025). The current study fills this gap by implementing the information-processing theory to describe the process by which marriage attitudes of unmarried women are influenced by Weibo marriage comments.

### 2.2 Elaboration likelihood model

The ELM states that attitude change occurs through two different processing routes. The central route requires careful argument processing, while the peripheral route occurs through heuristic cues, such as the characteristics of the source (Petty and Cacioppo, 1986). The processing of information is more likely to lead to a lasting attitude change. The peripheral route is adopted when the processing is low. This low processing ability is typically due to lacking motivation, and/or the ability to process the content. Thus, attitude change is more likely to depend on simple cues, as opposed to containing a detailed examination of the content of the message (Wagner and Petty, 2022; Hou, 2025).

In the ELM, two major components are source credibility and argument quality. The argument is the quality of the argument and the logic, which is the overall quality of the argument (Bhattacherjee and Sanford, 2006). The argument quality, which is evident through the Weibo comments, is the level of logical comments that are persuasive, and informative regarding marriage. Source credibility is the information source, and it is the extent to which the source is knowledgeable, and trusted (Ismagilova et al., 2020). On Weibo, source credibility is the level of qualitative informative comments, and the trust of the comment posters on marriage.

Research suggests that source credibility and argument quality play a significant role in shaping attitudes in online environments. These studies demonstrate the importance of traits of source credibility in shaping consumer attitudes and behaviors in the online space (Sparks et al., 2013; Belanche et al., 2021). Studies employing the ELM in a social media context have also established that argument quality and source credibility are important for attitude change in environments that generate user content such as online reviews, health misinformation, and electronic word-of-mouth (Cheung et al., 2012). Moreover, the persuasive power of the components of a message is not a random phenomenon: the valence or the stance of the persuasive message affects the degree to which stronger arguments change the position being advocated in the message and further, higher source credibility is suggested to enhance persuasive impact in online environments (Inuzuka et al., 2023; Javed et al., 2024). On Weibo, negative comments in marriage-related posts often reflect domestic violence, gender and job discrimination, and marriage costs to women (Cao, 2025). Negative valence in online reviews is most likely to shape attitudes, especially when the negativity is coupled with quality and credibility (Lee et al., 2008). In the context of a largely negative information environment, source credibility and argument quality are most likely to strengthen the impact of negativity and, in turn, would most likely lead to negative attitudes toward marriage.

Furthermore, research that combines ELM with behavioural intention models has shown direct effects of argument quality and source credibility on behavioural intention via routes that do not depend on attitudinal mediation (Cyr et al., 2018). Thus, in the context of negative marriage discourse, exposure to well-developed and credible anti-marriage arguments may trigger cognitive elaboration that negatively affects marriage intention—either by increasing the cognitive accessibility of negative marriage consequences or by increasing perceived dangers related to marrying (Hornikx, 2024).

### 2.3 Theory of planned behaviour

According to the TPB, behavioural intention is influenced by three criteria; attitude, subjective norms and perceived behaviour control (Ajzen, 1991). Attitude is the extent of an individual’s thought of either the positive or negative impacts of executing the behaviour; subjective norm is the social pressure from the significant people in one’s life concerning the behaviour. PBC is the perceived lack of or perceived presence of the resources needed to execute this behaviour and the absence of or presence of the constraints related to this behaviour.

TPB has been extensively researched to predict the intention toward marriage and improved fertility. One of the studies focused on the applicability of the TPB framework to reproductive decision-making, pointing out that the intentions toward creating a family have been shaped by attitudes, social norms and perceived control (Ajzen and Klobas, 2013). Empirical studies that have been carried out in the European context have indicated that the perceived behaviour control has a considerably strong influence on the intention to increase fertility as it reflects on the constraints of the economy and the constraints of the available institutions in the decisions about the family formation (Matera et al., 2023). Among the studies conducted in the Chinese context, research on fertility objectives of young people, using the TPB framework, provided a basis for prediction of the subjective norms due to the nature of the collectivism culture (Wang et al., 2025).

Positive or negative evaluations of the thought of marriage are what attitude means in a marriage context. A subjective norm can be the social influences, either positive or negative, that come from family, peers, and/or a partner regarding their views toward marriage. Looking at the role of marriage in the Chinese social system, it is both a personal and family choice that can impact family standing and responsibilities to descendants (Koçak and Mouratidis, 2024). PBC looks at how easy or tough a marriage is. Some of these things may include the financial, housing, and emotional readjustment of the marriage (Xie and Hong, 2022).

### 2.4 Mediating role of marriage attitude

This study combines elements of both the ELM and the TPB because both theories provide their own interpretation of attitudes and can be used theoretically together. Within the ELM, attitudes represent the way that individuals respond when confronted with persuasive information. This response is the result of the way that the individual has either centrally or peripherally construed the characteristics of that persuasive information (Petty and Cacioppo, 1986). Within the TPB, attitudes represent a person’s overall positive or negative assessment of the performance of a specific action (Ajzen, 1991). Despite the differences of these two theories and their origins, it has been found that distinct social attitudes, which have been formed and the processed with information, can help build specific attitudes which, in turn, can predict behaviour intentions (Zhao et al., 2020). This study also found that the attitudes that apply to a specific behaviour are based on the beliefs that are most prominent regarding that behaviour. When information is persuasive, these beliefs can be reshaped, which can subsequently change behavioural attitudes (Fishbein and Ajzen, 2011). In the current study, the persuasive information is negative comments regarding marriage and the behaviour that is observed (in the study) is the change in the attitudes of women regarding the beliefs surrounding marriage (i.e., the ideas surrounding marriage as a financial strain, a hindrance to career development, and a form of gender inequality).

This pathway indicates that attitudes toward marriage could mediate how Weibo comment characteristics relate to marriage intention. The ELM describes how attitudes are formed through the quality of arguments and the credibility of the sources that process information. The TPB describes how attitudes toward something will subsequently influence one’s intention to act. The communication literature describes how the integration of ELM and behavioural models demonstrates this meditative relationship through different contextual factors such as consumer behaviour, technology adoption, and health communication (Cyr et al., 2018; Pillai and Sivathanu, 2020; Ragab, 2022). In this case, the argument quality and source credibility of negative Weibo comments surrounding marriage may influence the marriage attitudes, subsequently impacting marriage intentions.

### 2.5 Moderating role of personal relevance

Personal relevance is when an individual interprets information in terms of how it relates to their convictions, needs, or goals. Personal relevance is a highly significant modulatory component in the ELM (Petty and Cacioppo, 1986; Celsi and Olson, 1988). The ELM indicates that personal relevance acts as the principal modulatory mechanism in the connections between the quality of arguments, source credibility, and attitudes (Huete-Alcocer, 2017; Verma and Yadav, 2021). Personal relevance steers the process of deeper evaluation and source credibility leading to an attitude that is long-lasting, and also leads individuals to more readily engage in central processing (Petty and Cacioppo, 2012). Personal relevance is also shown to encourage peripheral processing and attitude change as a result of more shallow processing.

**Figure 1:**
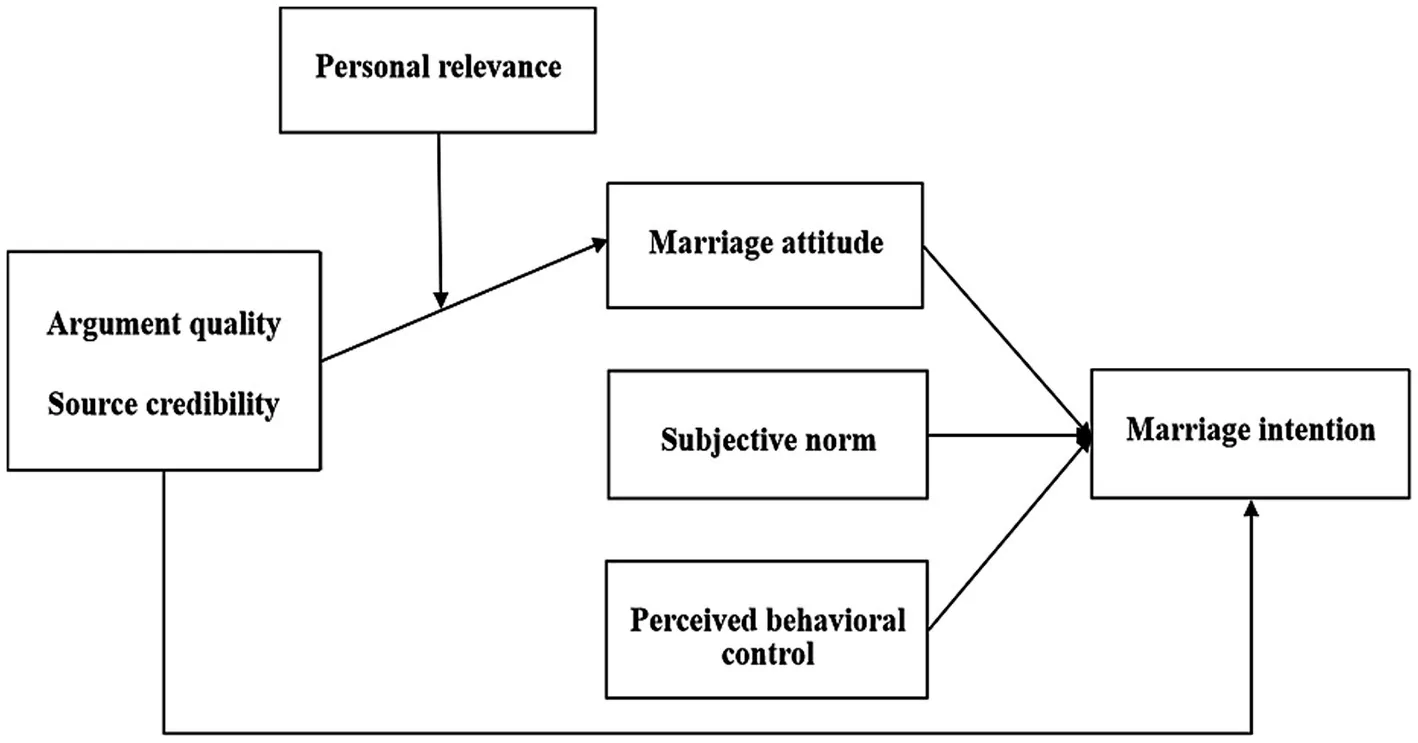
Conceptual framework of the study.

In light of Weibo marriage comments, the comments of (the) single women that interpret marriage related comments to that Weibo user’s personal life objectives, values, or life goals perhaps exhibit increased levels of deep processing, Weibo comments, to a greater extent than the average user, with greater scrutiny to the commenting logic and commenting persuasiveness and objectivity versus the average user or (the) average respondent. Such deep processing may also result increased source and argument effects. Accordingly, the following hypotheses are proposed (Figure 1):

H_1_: Argument quality of Weibo marriage comments is negatively related to marriage attitude.

H_2_: Source credibility of Weibo marriage comments is negatively related to marriage attitude.

H_3_: Argument quality of Weibo marriage comments is negatively related to marriage intention.

H_4_: Source credibility of Weibo marriage comments is negatively related to marriage intention.

H_5_: Marriage attitude is positively related to marriage intention.

H_6_: Subjective norms are positively related to marriage intention.

H_7_: PBC is positively related to marriage intention.

H_8_: Marriage attitude mediates the relationship between argument quality and marriage intention.

H_9_: Marriage attitude mediates the relationship between source credibility and marriage intention.

H_10_: Personal relevance moderates the relationship between argument quality and marriage attitude, such that the relationship is stronger when personal relevance is high.

H_11_: Personal relevance moderates the relationship between source credibility and marriage attitude, such that the relationship is stronger when personal relevance is high.

## 3 Methods

### 3.1 Research design and participants

This study employed a cross-sectional correlational design. Participants were recruited using a multi-stage sampling procedure across five levels: province, city, district, street, and community. Guangdong Province and Guangzhou City were purposively selected for their high urbanisation, social media penetration, and large population of unmarried women, representing a context where traditional and modern marriage values actively coexist (Ji, 2015). Tianhe District was randomly selected from 11 districts, Huangcun Street from 21 streets, and four communities (Huangcunxi, Kangcheng, Jiangxia, and Longbu) from nine communities.

The criteria for inclusion required participants to be: (a) female, (b) aged at least 20, (c) never married, and (d) interacted with marriage-related Weibo comments. Screening questions at the start of the survey helped with filtering out those who were not eligible. After sending the survey via the community’s official resident WeChat group, the data collection began (March 20 to April 20, 2023). Self-administered, electronic questionnaires were the means to accomplish data collection. We expect to have some level of self-selection bias because of the community WeChat groups, but considered this the best option considering how the community WeChat groups in community of urban Guangzhou serve as a main digital communication structure with nearly total coverage among the residents. Also, the WeChat-based questionnaires were preceded by multi-stage clustering of the geographic units (district, street, community), assuring the stratification of the sampling frame and not just a matter of some convenience sampling. We confirmed each of the respondents had previously interacted with marriage-focused comments on Weibo to be sure of consistency between the recruitment channel (WeChat) and the study context (Weibo). We also confirmed via intended consent to be sure respondents agreed to enter the lottery for 50 CNY.

Of 498 responses were received and 125 were eliminated. of these, 63 were eliminated based on exclusion criteria, 44 were classified as careless responders (indicated by rapid completion and straight-lining), and 18 were determined to be multivariate outliers based on Mahalanobis distance (*p* < 0.01). The final sample consisted of 373 participants (effective rate: 74.9%). The minimum sample requirement using G*Power 3.1 (7 predictors, *f*^2^ = 0.15, *α* = 0.05, power = 0.95) was 153 and with the Hair et al. (2021) 10-times rule for PLS-SEM, it was 60. The 373 sample was greater than both minimum sample sizes.

Regarding demographics, 180 participants (48.3%) were aged between 20 and 24, 106 (28.4%) were aged between 25 and 29, 76 (20.4%) were aged between 30 and 34 and the remaining 11 (2.9%) were aged 35 or above. Concerning education level, 150 (40.2%) had a bachelor’s degree, 143 (38.3%) a specialist’s degree, 31 (8.3%) had completed secondary education, 27 (7.2%) had completed a master’s degree, and 22 (5.9%) had junior high education.

### 3.2 Instruments

Before scaling, the participants were shown five marriage-related comments taken from Weibo, so they could operate using a common benchmark. As noted in other scholarship, offering respondents contextual situational examples before a questionnaire enhances memory for the study’s relevant context, therefore increasing accuracy (Payton and Gould, 2022; Velykoivanenko et al., 2024). Other studies focusing on the spread of information on the internet have adopted the same technique (Chen et al., 2021).

The start of 2022 saw Weibo’s hot search topic “The increased likelihood of women not marrying throughout their lives is worth reflecting on,” an article from the Guangming Daily published on Weibo, receive 94,000 comments. Searching/discussing/sharing popular topics is how hot searches are created. They reflect the most popular interests on Weibo (Cui and Kertész, 2023). We selected intense comments from the top 5 sections with the most likes and replies. Intense comments are the most published opinions and/or popular comments. These types of comments are the most published opinions and/or popular comments and offer the most varied opinions on the topic (Porten-Cheé and Eilders, 2020).

The comments we selected were largely negative, with complaints about issues such as domestic violence, violence at the workplace, financial issues, and the lack of balance between one’s own goals and society’s pressure to get married. These comments reflect the complaining and negative comments on Chinese social media (He et al., 2024). This close alignment indicates that the comments we selected were the real comments from Weibo, not something we carefully created to show a negativity bias.

The questionnaire consisted of seven multi-item scales, all rated on 7-point Likert scales ranging from 1 (strongly disagree) to 7 (strongly agree). Argument quality and source credibility were each measured with 4 items adapted from established scales (Bhattacherjee and Sanford, 2006). Personal relevance was measured with 4 items adapted from prior research (Kang et al., 2014). Marriage attitude, subjective norms, PBC, and marriage intention were measured with 5, 4, 4, and 4 items, respectively, based on the questionnaire construction guidelines of the TPB (Ajzen, 2002, 2006). A pilot study involving 40 participants was conducted prior to the main survey, and all scales demonstrated acceptable internal consistency, with Cronbach’s *α* values above 0.70.

### 3.3 Data analysis

Harman’s single factor test was employed to assess common method bias (CMB); the unrotated factor accounted for 31.16% of the variance (below 40%), suggesting there is no threat of CMB procedural measures such as anonymity and item separation. A full collinearity test was also performed; all VIFs were below 3.3, which also suggests common method bias is unlikely to affect the results (Kock, 2015).

Data were analysed using SmartPLS 4.0 and the partial least squares (PLS) approach in structural equation modelling. PLS-SEM was chosen due to the presence of mediation and moderation in the model, the partially exploratory nature of the theoretical integration, and because PLS-SEM does not assume multivariate normality (Hair et al., 2021). The analysis was undertaken in two stages: (1) measurement model for reliability (outer loadings > 0.708; CR > 0.70), convergent validity (AVE > 0.50), and discriminant validity (HTMT < 0.85); and (2) structural model for path coefficients, *R*^2^, *f*^2^, and *Q^2^* using bootstrap resampling (5,000 iterations). Mediation was assessed via specific indirect effects, and moderation via interaction terms and simple slope analysis.

## 4 Results

### 4.1 Measurement model assessment

The hypothesized seven-factor measurement model was evaluated using a confirmatory PLS-SEM approach based on the theoretical foundations of the TPB (Ajzen, 2002, 2006) the ELM (Bhattacherjee and Sanford, 2006). Although exploratory procedures such as parallel analysis may suggest alternative factor solutions, the purpose of the present study was not scale development but the confirmation of theoretically specified constructs. Therefore, the seven-factor structure was retained and assessed through established reliability and validity criteria recommended for PLS-SEM (Hair et al., 2021).

Indicator reliability, internal consistency reliability, convergent validity and discriminant validity were tested for the measurement model. As seen in Table 1, all 25 items demonstrated satisfactory indicator reliability, with outer loadings ranging from 0.752 to 0.929, exceeding the recommended threshold of 0.708. Therefore, no items were removed due to inadequate loading. Internal consistency reliability was supported, as Cronbach’s alpha values (range: 0.867–0.933), rho_A values (range: 0.869–0.938), and composite reliability values (range: 0.909–0.950) all exceeded the recommended threshold of 0.70 and remained within the acceptable range (≤ 0.95) (Hair et al., 2021). Convergent validity was confirmed, as all AVE values exceeded the recommended threshold of 0.50 (range: 0.715–0.790).

**Table 1:** Measurement model assessment.

Construct	Item	Loading	*α*	*ρ*A	CR (*ρc*)	AVE
Argument quality	AQ_1	0.803	0.877	0.880	0.916	0.731
	AQ_2	0.852				
	AQ_3	0.881				
	AQ_4	0.881				
Source credibility	SC_1	0.842	0.889	0.894	0.923	0.751
	SC_2	0.859				
	SC_3	0.898				
	SC_4	0.866				
Personal relevance	PR_1	0.840	0.867	0.869	0.910	0.716
	PR_2	0.883				
	PR_3	0.850				
	PR_4	0.809				
Marriage attitude	AT_1	0.929	0.933	0.938	0.950	0.790
	AT_2	0.827				
	AT_3	0.864				
	AT_4	0.905				
	AT_5	0.917				
Subjective norm	SN_1	0.874	0.867	0.887	0.909	0.715
	SN_2	0.752				
	SN_3	0.869				
	SN_4	0.881				
perceived behavioral control	PBC_1	0.882	0.910	0.923	0.936	0.786
	PBC_2	0.887				
	PBC_3	0.899				
	PBC_4	0.878				
Marriage intention	IN_1	0.908	0.898	0.901	0.929	0.767
	IN_2	0.839				
	IN_3	0.904				
	IN_4	0.849				

CR = composite reliability (ρc). All outer loadings >0.708. All CR values are between 0.70 and 0.950. All AVE values >0.50, confirming convergent validity.

Two approaches evaluated discriminant validity. First, the Fornell-Larcker criterion (Table 2) was met as the square root of AVE of each construct was greater than the correlations between the construct and other constructs. Second, all HTMT values (Table 3) were below the conservative benchmark of 0.85 (0.162–0.468; Henseler et al., 2015). Overall, these findings provide empirical support for retaining the proposed seven-construct measurement model.

**Table 2:** Discriminant validity—Fornell–Larcker criterion.

No.	Construct	1	2	3	4	5	6	7
1	AQ	**0.855**						
2	MA	−0.374	**0.889**					
3	MI	−0.415	0.416	**0.876**				
4	PBC	−0.220	0.267	0.307	**0.887**			
5	PR	0.196	−0.322	−0.245	−0.154	**0.846**		
6	SC	0.409	−0.382	−0.420	−0.153	0.249	**0.866**	
7	SN	−0.288	0.425	0.392	0.317	−0.204	−0.194	**0.846**

AQ = argument quality, MA = marriage attitude, SC = source credibility, MI = marriage intention, SN = subjective norm, PBC = perceived behavioral control, PR = personal relevance. Diagonal values (bold) represent the square root of AVE. Off-diagonal values represent inter-construct correlations.

**Table 3:** Discriminant validity—HTMT ratio.

Construct	AQ	MA	MI	PBC	PR	SC
MA	0.409					
MI	0.468	0.454				
PBC	0.236	0.287	0.331			
PR	0.224	0.356	0.275	0.174		
SC	0.460	0.412	0.466	0.162	0.279	
SN	0.331	0.467	0.437	0.353	0.230	0.217

AQ = argument quality, MA = marriage attitude, SC = source credibility, MI = marriage intention, SN = subjective norm, PBC = perceived behavioral control, PR = personal relevance. All HTMT values <0.85, indicating adequate discriminant validity.

### 4.2 Structural model assessment

**Figure 2:**
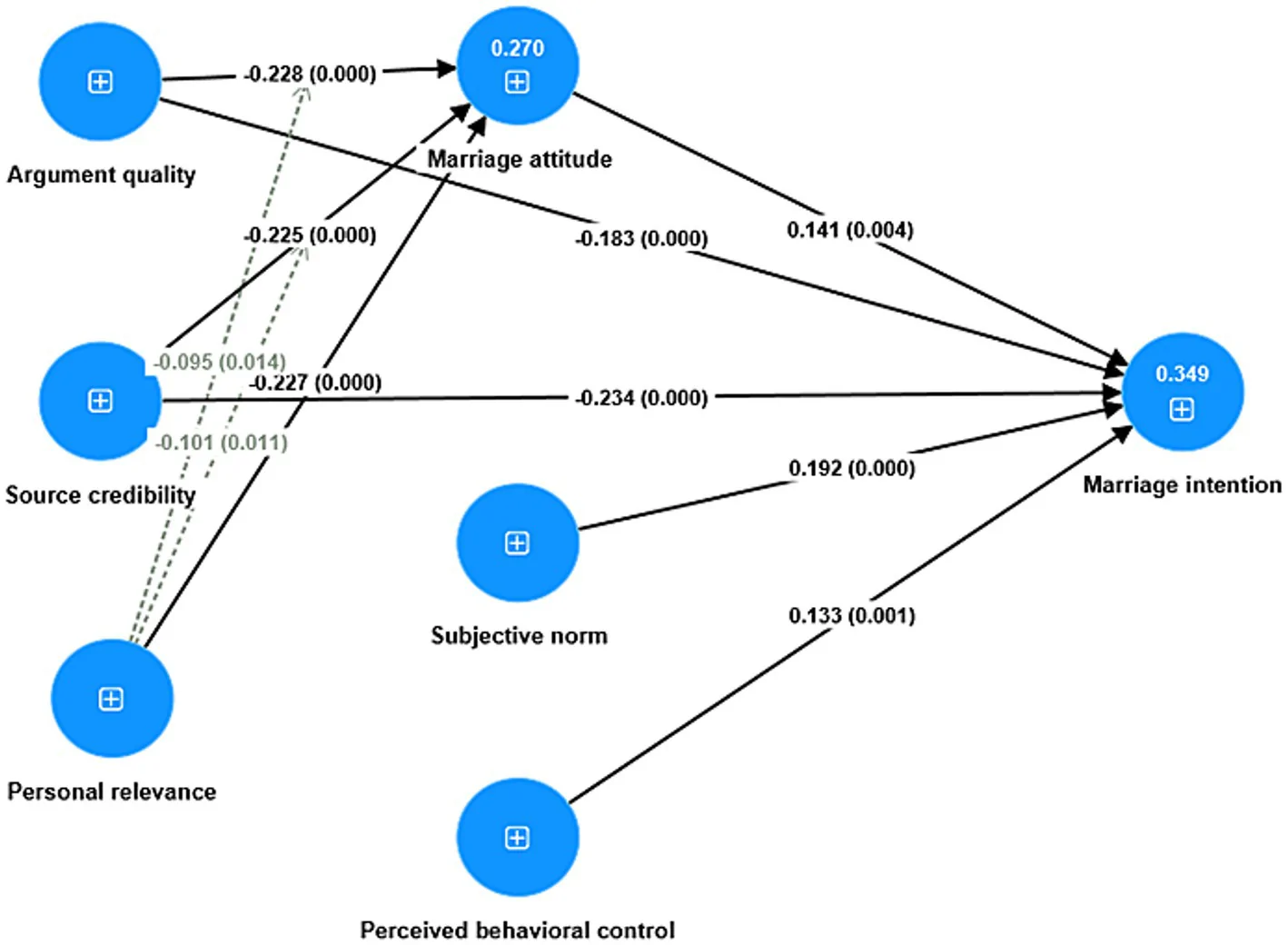
Structural model of the study constructs.

Collinearity was assessed prior to hypothesis testing. All variance inflation factor (VIF) values were below 5.0, indicating no multicollinearity issues in the structural model. The coefficient of determination (*R*^2^) for marriage intention was 0.349, indicating that the exogenous variables explained 34.9% of the variance in marriage intention (Figure 2). The *R*^2^ for marriage attitude was 0.270, indicating 27.0% of variance explained. Both values exceeded threshold of 0.26 for substantial explanatory power (Cohen and Cohen, 2013; Table 4).

**Table 4:** Structural model assessment (*R*^2^, *Q*^2^, *f*^2^, and VIF).

Construct	*R* ^2^	*R*^2^ adj.	*Q* ^2^	*f*^2^ (MI)	*f*^2^ (MA)	VIF(MI)	VIF(MA)
MI	0.349	0.340	0.260	–	–	–	–
MA	0.270	0.260	0.206	0.021	–	1.444	–
AQ	–	–	–	0.039	0.058	1.326	1.225
SC	–	–	–	0.065	0.055	1.296	1.254
SN	–	–	–	0.043	–	1.312	–
PBC	–	–	–	0.024	–	1.151	–
PR	–	–	–	–	–	–	1.084

AQ = argument quality, MA = marriage attitude, SC = source credibility, MI = marriage intention, SN = subjective norm, PBC = perceived behavioral control, PR = personal relevance.

Predictive relevance (*Q*^2^) was assessed using the blindfolding procedure. *Q*^2^ values for both marriage intention and marriage attitude were above zero, confirming the model’s predictive relevance. The effect sizes (*f*^2^) indicated that all exogenous variables had small effects on the endogenous variables, with values ranging from 0.021 to 0.065 (Cohen and Cohen, 2013).

### 4.3 Hypothesis testing: direct effects

The bootstrap analysis (5,000 resamples) revealed that all direct path relationships were statistically significant. Table 5 presents the path coefficients for the direct effects.

**Table 5:** Direct effects path coefficients.

Relationship	*β*	*SD*	*t*	*p*	95% CI LL	95% CI UL
AQ → MA (H_1_)	−0.228	0.047	4.805	<0.001	−0.322	−0.136
SC → MA (H_2_)	−0.225	0.048	4.659	<0.001	−0.318	−0.127
AQ → MI (H_3_)	−0.183	0.051	3.604	<0.001	−0.283	−0.083
SC → MI (H_4_)	−0.234	0.046	5.113	<0.001	−0.324	−0.145
MA → MI (H_5_)	0.141	0.052	2.693	0.004	0.038	0.241
SN → MI (H_6_)	0.192	0.051	3.734	<0.001	0.091	0.292
PBC → MI (H_7_)	0.133	0.044	3.057	0.001	0.049	0.222

AQ = argument quality, MA = marriage attitude, SC = source credibility, MI = marriage intention, SN = subjective norm, PBC = perceived behavioral control.

Argument quality was significantly and negatively related to marriage attitude (beta = −0.228, *t* = 4.805, *p* < 0.001, 95% CI [−0.322, −0.136]), supporting H_1_. Source credibility was also significantly and negatively related to marriage attitude (beta = −0.225, *t* = 4.659, *p* < 0.001, 95% CI [−0.318, −0.127]), supporting H_2_.

Regarding the direct effects on marriage intention, argument quality had a significant negative effect (beta = −0.183, *t* = 3.604, p < 0.001, 95% CI [−0.283, −0.083]), supporting H_3_. Source credibility also had a significant negative effect on marriage intention (beta = −0.234, *t* = 5.113, *p* < 0.001, 95% CI [−0.324, −0.145]), supporting H_4_.

Among the TPB variables, marriage attitude (beta = 0.141, *t* = 2.693, *p* = 0.004, 95% CI [0.038, 0.241]), subjective norms (beta = 0.192, *t* = 3.734, *p* < 0.001, 95% CI [0.091, 0.292]), and PBC (beta = 0.133, *t* = 3.057, *p* = 0.001, 95% CI [0.049, 0.222]) were all positively and significantly related to marriage intention, supporting H_5_, H_6_, and H_7_, respectively.

### 4.4 Mediation analysis

The mediating effect of marriage attitude was examined through specific indirect effects. The indirect effect of argument quality on marriage intention through marriage attitude was significant (beta = −0.032, *t* = 2.284, *p* = 0.022, 95% CI [−0.063, −0.008]), with the confidence interval excluding zero. Likewise, the indirect effect of the source credibility on marriage intention through marriage attitude was significant (beta = −0.032, *t* = 2.347, *p* = 0.019, 95% CI [−0.060, −0.008]).

The direct and indirect effects were negative in both pathways, which suggested that marriage attitude was a complementary mediator, thus supporting H_8_ and H_9_. This complementary mediation pattern suggests that marriage attitude is one of several pathways through which characteristics of Weibo comments influence marriage intention (Table 6).

**Table 6:** Mediation effects (specific indirect effects).

Indirect path	*β*	*t*	*p*	95% CI LL	95% CI UL
AQ → MA → MI (H_8_)	−0.032	2.284	0.022	−0.063	−0.008
SC → MA → MI (H_9_)	−0.032	2.347	0.019	−0.060	−0.008

AQ = argument quality, MA = marriage attitude, SC = source credibility, MI = marriage intention.

### 4.5 Moderation analysis

Personal relevance significantly moderated the relationship between argument quality and marriage attitude (beta = −0.095, *t* = 2.186, *p* = 0.014, 95% CI [−0.178, −0.009], *f*^2^ = 0.010), supporting H10. Personal relevance also significantly moderated the relationship between source credibility and marriage attitude (beta = −0.101, *t* = 2.284, *p* = 0.011, 95% CI [−0.182, −0.011], *f*^2^ = 0.011), supporting H_11_ (Table 7).

**Table 7:** Moderating effects of personal relevance.

Interaction	*β*	*t*	*p*	95% CI LL	95% CI UL	*f^2^*
PR × AQ → MA (H_10_)	−0.095	2.186	0.014	−0.181	−0.024	0.010
PR × SC → MA (H_11_)	−0.101	2.284	0.011	−0.186	−0.029	0.011

AQ = argument quality, MA = marriage attitude, SC = source credibility, PR = personal relevance.

**Figure 3:**
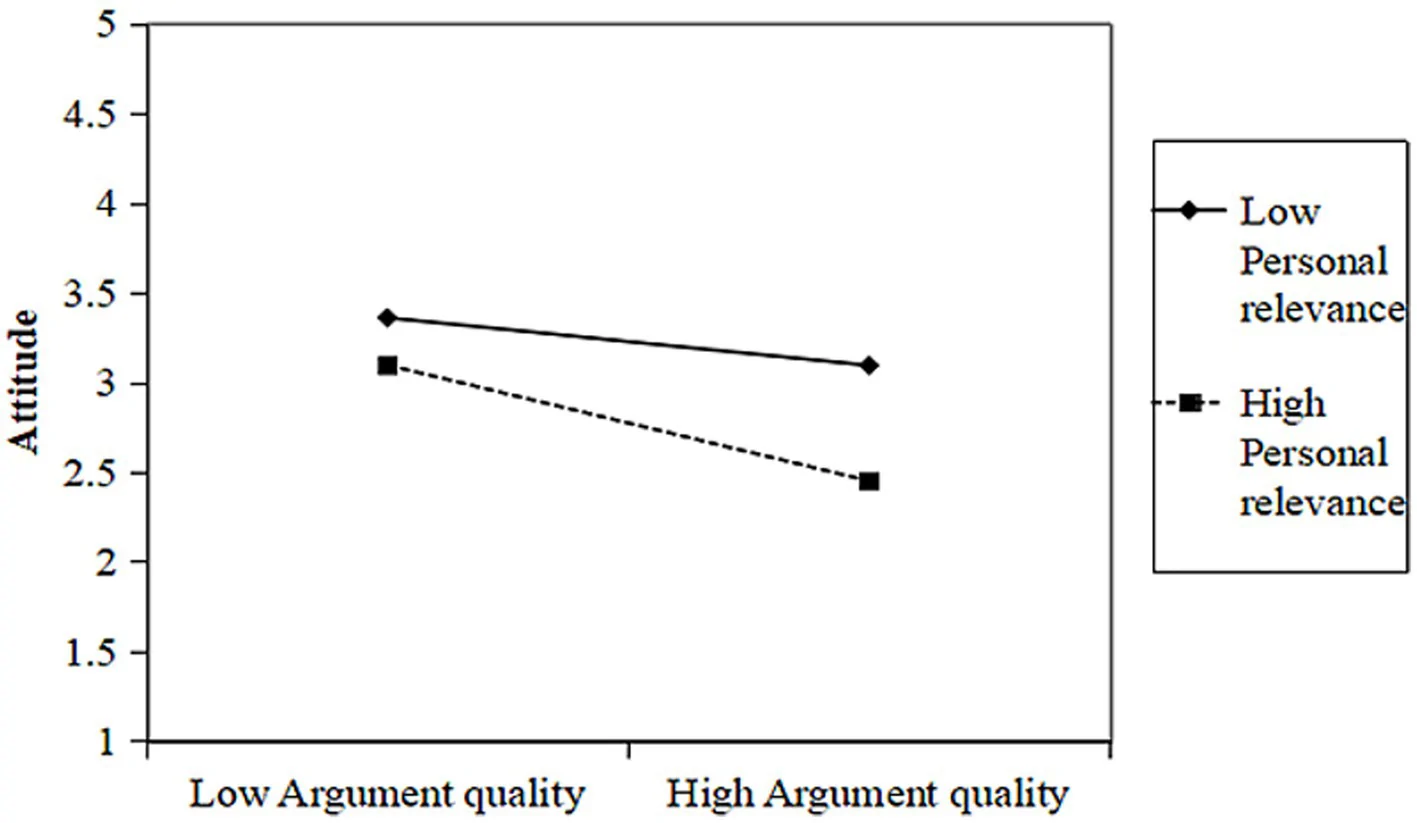
Interaction effect of personal relevance and argument quality on marriage attitude.

**Figure 4:**
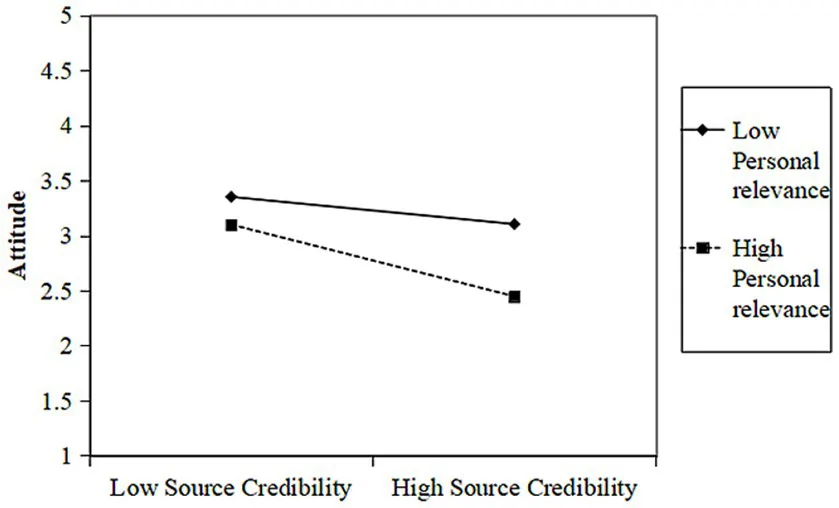
Interaction effect of personal relevance and source credibility on marriage attitude.

Simple slope tests showed that the negative associations between argument quality and marriage attitude, and source credibility and marriage attitude, were greater when personal relevance was perceived to be high, than when perceived personal relevance was low. The interaction plots revealed that the lines labelled high personal relevance were steeper than those labelled low personal relevance, which confirmed that when single women find comments about marriage highly relevant to their personal situation, argument quality and source credibility have a greater persuasive influence on their attitudes toward marriage (Figures 3, 4).

## 5 Discussion

This research combined the ELM and TPB to explore the effect of Weibo marriage comments on Chinese single women’s marriage attitudes and intentions. The results confirmed the 11 hypotheses. The next sections interpret the important findings in the context of existing research and their theoretical and practical implications.

### 5.1 Effects of Weibo comment characteristics on marriage attitude

The quality of the argument (*β* = −0.228) and the credibility of the source (*β* = −0.225) of Weibo marriage comments were negatively linked to marriage attitude, as predicted in H1 and H2. The results contribute to the application of the ELM to the field of marriage-related social media messages, an area that has not previously received much attention in the persuasion research. The negative effects of argument quality and source credibility are likely due to the mostly negative nature of the stimulus comments. According to the ELM, argument quality and source credibility are valence-neutral moderators that enhance persuasion in the direction of the advocated message (Petty and Cacioppo, 1986, 2012). This interpretation is consistent with meta-analytic evidence demonstrating that negative online content exerts disproportionate influence on attitude formation when perceived as high in quality and credibility (Lee et al., 2008; Ma and Atkin, 2017; Liang et al., 2021).

Interestingly, the effect sizes of argument quality and source credibility were nearly equal (*β* = −0.228 and −0.225, respectively), implying that both central and peripheral processing are active in this situation. This result contrasts with previous ELM research in consumer and health settings, which found argument quality tends to prevail under high-involvement conditions (Petty and Cacioppo, 1984). This may be due to the nature of the Weibo information environment: marriage comments are short, user-generated, and appear in an information stream that scrolls quickly, limiting the depth of systematic processing even for highly motivated users. This explanation aligns with recent studies in the ELM literature that have found a blurring of central and peripheral processing in social media environments, as a result of the multi-cue, mixed-media nature of information presented (Miller et al., 2004).

The results also add to the burgeoning research on social media and marriage attitudes in East Asia. Although previous studies have reported a trend toward declining marriage rates and the rise of singlehood for Chinese women (Raymo et al., 2015), few empirical studies have investigated how online discourse affects attitudinal change (Yu and Tian, 2023). This research offers evidence that social media comments operate as persuasive cues that can systematically influence marriage attitudes via specific information-processing routes.

### 5.2 Direct and indirect effects on marriage intention

Argument quality (*β* = −0.183) and source credibility (*β* = −0.234) had significant negative direct effects on marriage intention in addition to the indirect effects through marriage attitude. The parallel mediation model suggests that marriage attitude is just one pathway by which Weibo comments impact marriage intention (Zhao et al., 2010). The significant direct effects indicate negative marriage comments may impact intention in non-attitudinal ways. Imagable negative marriage comments may increase the cognitive accessibility of negative marriage exemplars, increasing the salience of negative marriage outcomes when considering marriage (Zhang et al., 2025). Further, negative marriage comments with high source credibility may increase perceived risk of marriage, which in turn decreases marriage intention (without changing attitudes).

Although the indirect effects of argument quality and source credibility on marriage intention through marriage attitude were statistically significant, they were small (*β* = −0.032 for both pathways), particularly compared to the direct effects of argument quality (*β* = −0.183) and source credibility (*β* = −0.234) on marriage intention. This pattern needs to be interpreted with caution. The relatively small indirect effects suggest that while marriage attitude is a statistically significant mediator, it plays a weaker role as a transmission mechanism than do direct effects. The complementary mediation pattern, in which both direct and indirect effects are significant and in the same direction, implies that the effects of Weibo comment characteristics on marriage intention are largely non-attitudinal, with attitude change being a subsidiary mechanism (Zhao et al., 2010). The direct effects may reflect cognitive accessibility of negative marriage exemplars (Zhang et al., 2025), perceived risk of marriage, and affect that does not translate into deliberative attitude formation. This finding is in line with dual-process theories that suggest behavioural intentions can be formed through both deliberative (attitude-mediated) and impulsive (direct) routes [Reflective-Impulsive Model, RIM] (Strack and Deutsch, 2004). Future studies using more specific mediators, such as perceived marriage risk, negative affect, or marriage self-efficacy, might reveal other indirect effects that are not specified in the current model.

The TPB variable with the largest significant impact on marriage intention was subjective norms (*β* = 0.192), followed by marriage attitude (*β* = 0.141) and PBC (*β* = 0.133). The high salience of subjective norms reflects the persistence of family norms in the Chinese collectivist culture (Zhong and Wilkinson, 2025). This observation is in line with recent TPB studies of family formation intentions in collectivist and East Asian samples, where subjective norms consistently prove to be significant predictors alongside or surpassing personal attitude (Farrukh et al., 2019). But the relatively small size of the subjective norms effect (*β* = 0.192) relative to its strong historical prominence in Chinese family formation suggests that social norms may be shifting among young urban women. This finding is in line with recent studies that show the increasing preference for autonomy and individual fulfilment among Chinese millennials and Generation Z, and the shift from marriage as a family duty to marriage as a personal choice (Wang et al., 2025). The loss of normative pressure and the rise of individualism in modern Chinese society contribute to the ambivalent psychological context reflected in the moderate levels of all the variables in this study. The strong positive association between PBC and marriage intention (*β* = 0.133) suggests that perceptions of practical feasibility (such as financial, housing and emotional readiness) is an important factor. This is consistent with research, which suggests that economic factors are a major obstacle to marriage for young Chinese adults, especially in first-tier cities such as Guangzhou, where the cost of living and housing is prohibitively expensive (Xie and Hong, 2022).

It is important to note that the model accounted for 34.9% of variance in marriage intention and 27.0% in marriage attitude. These are above the threshold for significant explanatory power in behavioural science research (Cohen and Cohen, 2013), but also suggest that two-thirds of the variance in marriage intention is unaccounted for by the proposed model. This is not surprising because the decision to marry is a complex process influenced by a range of factors in addition to those examined in this study, including factors such as personality, relationship status and quality, parental marital experience, economic, and housing affordability (Raymo et al., 2015; Xie and Hong, 2022). The relatively low R^2^ values, therefore, reflect a trade-off inherent in parsimony: the integrated ELM-TPB model trades off predictive power for theoretical consistency. Future studies might build on the model by including additional measures such as perceived marriage risk, experience in dating and courting, or particular economic factors, to increase variance explained.

### 5.3 Moderating role of personal relevance

Personal relevance moderated the effects of argument quality and source credibility on marriage attitude, with the negative effects becoming more pronounced at higher levels of personal relevance (H_10_ and H_11_). These results support the ELM’s central tenet that personal relevance boosts the likelihood of message elaboration, thereby enhancing the effects of the message on attitude change (Petty and Cacioppo, 1986; Petty et al., 2009). Yet, the moderating effects of personal relevance, measured by the effect sizes (*f*^2^) of the interaction terms, were small (*f*^2^ = 0.010 and 0.011) compared to the conventional effect size of 0.02 for a small effect (Cohen and Cohen, 2013). This must be admitted. One explanation is that the moderating role of personal relevance may be more pronounced on the persistence and resistance of attitudes, than on the initial development of attitudes (Petty et al., 2009). The present study has a cross-sectional design and only provides a static picture of attitude states; the moderating effects could be larger in longitudinal measures of attitude persistence. Also, the moderate mean levels of personal relevance (implying neither very high nor low levels of relevance) may have limited the moderator effect, resulting in weaker interaction effects in this study.

Despite the moderate effect sizes, this moderation effect has important theoretical and practical implications. The moderation effect suggests that single women who are in the process of engaging in decision-making for marriage may be the most vulnerable to the persuasive influence of Weibo’s negative marriage messages. This is in line with ELM findings that when people have a high degree of personal involvement they devote more elaborate cognitive processing, and as such are more influenced by argument quality and, under some circumstances, source credibility (Petty et al., 2009; Petty and Cacioppo, 2012; Javed et al., 2024). This suggests that media literacy may be most important for women in the active marriage decision-making period for policymakers and social platforms.

## 6 Conclusion

This study confirms that Weibo marriage comments operate as persuasive messages that reduce marriage attitudes and intentions among single Chinese women via specific information processing mechanisms. By linking ELM and TPB via the bridging factor of marriage attitude, this study identifies a two-pronged influence mechanism: characteristics of Weibo comments directly and indirectly influence marriage intention through attitude change, with personal relevance enhancing these effects by increasing cognitive elaboration. The key theoretical contribution is the showing that persuasion processes outlined by the ELM, based on laboratory-based experiments, succeed in the real-life, user-created information environment of Chinese social media. The combination with TPB also shows that social media-induced attitudes flow into intention in combination with culturally ingrained subjective norm and perceived behavioural control, offering a more holistic view of marriage decision-making among modern Chinese women.

These results have three practical implications. First, policy makers interested in marriage rates should recognise that social media play an important role in shaping attitudes toward marriage and may consider incorporating media literacy training into existing marriage-related interventions. Second, social media platforms should adopt content diversification approaches in their algorithms to ensure that users are exposed to a broader range of marriage-related perspectives rather than predominantly negative content. Third, local marriage education programs could address common concerns expressed through online social media communication, such as financial challenges and gender inequality, by providing evidence-based advice and resources.

## 7 Limitations and future research

There are several limitations in this study. First, the cross-sectional design limits causal inference; therefore, longitudinal and experimental studies are needed to examine the temporal relationship between social media exposure, marriage attitudes, and marriage intentions, as well as the stability of attitude changes over time. Second, this study was conducted among unmarried women from four communities in Tianhe District, Guangzhou. Although this context provides valuable insights into marriage decision-making among urban Chinese women, the findings may not be fully generalisable to unmarried women in other regions, such as second- or third-tier cities, rural areas, or populations with different socioeconomic and cultural backgrounds. Third, although the multi-stage sampling approach provided geographic guidance for community selection, the use of community-based WeChat groups for questionnaire distribution may have introduced self-selection bias, as individuals who are more active online may have been more likely to participate. Fourth, the sample was relatively young (48.3% aged 20–24) and highly educated (78.5% had a specialist degree or higher), which may limit the generalisability of the findings to older or less educated unmarried women. Future research should extend this model by examining other social media platforms (e.g., Douyin and Xiaohongshu), including different participant groups (e.g., unmarried men and older unmarried women), and applying the framework across diverse cultural and regional contexts to further assess the generalisability of the findings.

## Data Availability

The raw data supporting the conclusions of this article will be made available by the authors, without undue reservation.
